# Six-Year Molars

**Published:** 1873-01

**Authors:** L. W. Munhall


### Six-Year Molars.

BY L. W. MUNHALL.

Every dentist cannot but have observed what a large per cent. of these *most* important teeth are absent from the mouths of adults, and are constantly being sacrificed. This condition of affairs is attributable, as I think, to two causes: First, the ignorance and indifference of parents; and second, the ignorance and indifference of the dentist.

Respecting the first cause, I think the parent *almost* wholly to blame. I say *almost* wholly, because I think the dentist is partially in many cases, and largely in some.

As a profession, we are educators in a very important sense. What do the masses know of the advances made by the profession, except as the profession communicates such information to them? How often do we hear it said, "I thought that tooth to far gone to be filled!" "I didn't know a tooth that had ached could be filled!" and other statements of a kindred sort. And respecting this very matter under consideration, when the parent brings the child to the dentist to have the six-year molars extracted, because they ache, and are scolded for their carelessness in letting their teeth get in such a condition, is it not the almost universal reply, "I thought those were first teeth, and that others would come in when these were removed." The number of persons rightly informed concerning this matter is very few. Of course there is a large class that cannot be reached; but all who are our regular patrons may be fully instructed, as well, also, in a measure, those who are transient and irregular. No parent or guardian should be allowed to take a child from our offices without having their attention called to this matter, and, if need be, instructed concerning it.

And now, respecting the second cause: Did you ever hear of a dentist extracting one of these molars, and assigning as a reason why it might be removed without injury, that "the teeth are crowded, and if extracted, the other molars will move forward and occupy the space?" I have, and in a very great many instances; so often, indeed, that I am disposed to think it a very common thing for dentists to talk thus. The statement may be literally true, *per se*, in a great many cases; but a dentist who would give such an opinion must be either ignorant or indifferent. Let me give a case.

Herbert E——, aged 12, came to my office, and represented that he had been getting some teeth filled by a certain dentist; and, that he—the dentist—wanted to extract the inferior six-year molars; but that his father had objected, and directed him to come to me for my opinion in the matter. I made a diagnosis, and found the teeth very far gone with caries, and the pulps entirely devitalized. There were no indications of abscess or suppuration. The boy was an unusually bright one. He had a splendidly developed head, the frontal and temporal bones being noticeably prominent. The superior maxilla was of good size and shape; but for some reason the inferior was disproportionately small—the chin being very much retired, so much so, that the inferior incisors closed inside the superior fully three quarters of an inch. Of course the lower teeth were very much crowded; and, had these molars been extracted, there is no doubt but that the space thus made vacant would, in time, have been occupied by the other teeth, even as the dentist who wanted to extract them had said. I afterward removed the diseased bone, treated the teeth with applications of a solution of carbolic acid and tinct. of iodine, and filled them with gold. This was about a year ago. I saw the boy a few days since, and the teeth are doing fully as well as I could wish.

Respecting this case, I hold that by reason of the contracted condition of the inferior maxilla there was an especial reason for retaining these teeth; but there were good and sufficient reasons for retaining them, even though this condition did not exist, It is always perilous to the contour and symmetry of the maxilla to remove a permanent tooth during the period of bone development; therefore, these teeth, as well as all others, should be retained as long as possible.

There are some apparent difficulties to a universal practice of this sort, of which I shall mention but two.

First, There are certain systematic conditions that would seem to justify the practitioner in making no effort to exert his saving powers. Take, for instance, a case where the teeth are imperfect in their structure, where there seems to be almost an entire absence of bone-making material. By reason of this condition they decay rapidly, and are so frail and soft they could be cut to pieces easily with an excavator. Now, in a case like this, he might not hope to permanently save; yet he may almost always be able to retain the teeth until the period of physical development is complete. I shouldn't hesitate to try, even though the patient hadn't enough phosphorus in his system to keep him in toe nails.

The other difficulty is the inability and unwillingness of parents to remunerate the dentist for his services. We love to talk of the dentist as a benefactor. It's a very easy matter to be a benefactor when one is pecuniarily rewarded for it; but, I take it, that that member of our profession is a true benefactor who, rather than let a child suffer because of the ignorance or sordidness of its parent, would render it a service without remuneration. It is the province of the dentist to save, not destroy; and to prevent destruction as far as possible.

If this article will be the means of causing a single member of the profession to be more faithful to his trust, I shall be rewarded for the labor of preparing it, for this is the object had in view.

